# Variability and Influencing Factors of Suicidal Ideation in Adolescents—An Ecological Momentary Assessment Study

**DOI:** 10.1111/sltb.70149

**Published:** 2026-11-08

**Authors:** Tamara Großmann, Matthias Lühr, Nadine Bayer, Jörg M. Fegert, Verena Gindele, Sophie Köster, Sabine Müller, Miriam Rassenhofer, Christian Rauschenberg, Ulrich Reinighaus, Theresa Schockenhoff, Ulrike M. E. Schulze, Heike Speth, Nathalie Oexle

**Affiliations:** ^1^ Department of Psychiatry and Psychotherapy II University of Ulm and BKH Günzburg Günzburg Germany; ^2^ German Center for Mental Health (DZPG), Partner Site Mannheim‐Heidelberg‐Ulm Mannheim Germany; ^3^ Clinic for Child and Adolescent Psychiatry, Psychosomatics and Psychotherapy Ulm University Medical Center Ulm Germany; ^4^ Clinic for Child and Adolescent Psychiatry and Psychotherapy Ravensburg ZfP Südwürttemberg Ravensburg Germany; ^5^ Department of Public Mental Health, Central Institute of Mental Health, Medical Faculty Mannheim Heidelberg University Mannheim Germany; ^6^ Health Service and Population Research Department, Institute of Psychiatry, Psychology and Neuroscience King's College London London UK; ^7^ Department of Child and Adolescent Psychiatry and Psychotherapy Böblingen ZfP Calw Böblingen Germany

**Keywords:** adolescents, ecological momentary assessment, multilevel analysis, suicidal ideation, variability

## Abstract

**Objective:**

Recent research demonstrated considerable variability in suicidal ideation (SI) among adults. This study aimed to extend these findings to adolescents by examining the variability of SI and exploring temporal and contextual factors contributing to SI in adolescents.

**Method:**

We recruited 51 adolescents (aged 12–18 years) from three German inpatient child and adolescent psychiatric hospitals, who reported current passive and active SI via smartphone‐based ecological momentary assessment 7 times per day over 7 days. Variability statistics included intra‐class correlation coefficient (ICC), within‐person variance (WPV), mean square of successive differences (MSSD) and its root (RMSSD), and probability of acute change (PAC). Furthermore, we investigated whether temporal (time of day, day of week) and contextual factors (physical activity, being alone) contributed to SI using multilevel models.

**Results:**

All variability measures indicated substantial within‐person variability of SI between measurements. Multilevel analyses found significant effects of time of day, day of week, and physical activity on SI, whereas being alone showed no significant effect.

**Conclusions:**

These findings underline the importance of considering SI as a dynamic construct and emphasize the need for continuous, context‐sensitive assessment to guide tailored interventions that reduce suicide risk among adolescents.

## 1 Introduction

Suicide is one of the leading causes of death among adolescents worldwide (World Health Organization 2025). In 2024, 216 adolescents aged ≤ 19 years died by suicide in Germany (Destatis 2025). However, a larger proportion is affected by suicidal ideation (SI; Evans et al. 2005)—including thoughts, intentions, and plans—which is particularly prevalent among inpatient adolescents (Alqueza et al. 2023). Although suicide is a multifactorial phenomenon, SI is a strong risk factor for suicide (Favril et al. 2023).

Previous research (e.g., Coppersmith et al. 2023) suggested that SI can fluctuate both over short periods (e.g., within hours) and across longer durations (e.g., weeks). However, SI has traditionally been assessed retrospectively to capture long‐term fluctuations. Short‐term fluctuations have been studied less often, as they require ecological momentary assessment (EMA) involving a high number of daily assessments of individuals' everyday experiences (Trull and Ebner‐Priemer 2013). EMA was recently established as a promising method in suicide research, including studies on SI among adolescents (Czyz et al. 2018; Glenn et al. 2022).

Existing EMA research has highlighted the dynamic nature of SI and its short‐term fluctuations among adults, showing that SI can fluctuate within hours or even within minutes (Coppersmith et al. 2023; Kleiman et al. 2017). Although fluctuations in SI are assumed to be more pronounced among adolescents (Czyz et al. 2019), studies remain sparse (Czyz et al. 2019; Andrewes et al. 2024). For instance, Czyz et al. (Czyz et al. 2019), who conducted an EMA study among adolescents (1 prompt/day for 4 weeks after discharge), reported substantial variability in SI between days. However, daily assessments cannot capture fluctuations occurring within hours or minutes. Addressing this limitation, Andrewes et al. (Andrewes et al. 2024) used high‐frequency assessments (six daily prompts on 7 days) among 15–25‐year olds with borderline personality disorder (BPD) and demonstrated that SI fluctuates both within and across days. These findings highlight the importance of high‐frequency assessments for capturing the dynamics of SI among adolescents. Furthermore, only a few EMA studies in adults have differentiated between passive (i.e., thoughts about death or a wish to die) and active SI (i.e., explicit thoughts of ending one's life) (e.g., Hallensleben et al. 2018), and only one study has done so in adolescents (Weires et al. 2025), without examining SI variability. To the best of our knowledge, no prior study has used high‐frequency assessments while explicitly differentiating between passive and active SI to examine SI variability in adolescents.

Existing studies reporting short‐term variability in SI suggested that SI fluctuates over time and across situations; however, the temporal and contextual factors contributing to these fluctuations remain unclear. Previous research in adult and young adult populations has examined the influence of time of day as well as weekday patterns in suicide mortality. These studies reported an increased risk of suicide attempts during the late evening, as well as higher rates of suicide attempts and completed suicides in the early morning, followed by a decrease throughout the day (Freichel and O'Shea 2023; Akkaya‐Kalayci et al. 2017). Furthermore, Lee et al. (Lee et al. 2024) reported lower suicide incidences on weekends in Europe, with the highest incidence on Mondays—a pattern similarly observed among youth (Hatton et al. 2024; Kim et al. 2019). Beyond temporal factors, research has also shown that physical activity (PA) is associated with lower rates of mental health problems and SI (McMahon et al. 2017; Babiss and Gangwisch 2009). However, to our knowledge, no study has examined the influence of momentary PA on momentary SI. Further, studies indicated that spending most of one's time alone is associated with elevated suicide risk (McClelland et al. 2023; Heß and Weiss 2024). Specifically, a recent EMA study among young adults found that participants reported decreased momentary SI in moments when they were not alone (Brown and Scott 2025).

In summary, research examining short‐term variability of SI among adolescents remains limited, as studies using high‐frequency assessments and assessing both passive and active SI are rare. To address this gap, we conducted an EMA study with seven daily assessments to (1) examine the variability of passive/active SI and (2) investigate the influence of temporal (time of day, day of the week) and contextual factors (activity, social context) on passive/active SI. Based on previous literature described above, we hypothesized increased SI in the evening (H1), on Mondays (H2), during non‐physical activities (H3), and when adolescents are alone (H4).

## 2 Methods

### 2.1 Participants and Recruitment

This study is part of the EMIRA project (ecological momentary intervention to reduce suicide risk among adolescents), which aims to develop and evaluate a safety planning smartphone app for adolescents. To inform subsequent app development, we conducted an EMA study on adolescents' SI dynamics. Between October 2023 and October 2024, we recruited participants from three child and adolescent psychiatric hospitals in Southern Germany through direct approach by practitioners. Eligible adolescents were 12–21 years old, in inpatient treatment, had a history of SI, and had a safety plan (i.e., a personalized plan for coping with suicidal crisis). Adolescents were excluded if practitioners raised safety or feasibility concerns (e.g., acute suicide risk, cognitive impairment).

### 2.2 Study Procedure

Informed consent was obtained from all participants and, for minors, their legal guardians. The study design comprised a baseline assessment, followed by a 7‐day EMA phase with seven daily prompts, and a subsequent follow‐up assessment. After the baseline assessment, participants received instructions on completing the EMA prompts. Starting the next day, EMA prompts were delivered randomly between 7:00 a.m. and 9:00 p.m. to capture daytime experiences while minimizing disruptions to inpatient routines, particularly nighttime rest. Assessments had to be completed within 45 min, with at least 60 min between prompts. EMAs were conducted via MovisensXS on study‐smartphones (Android). For participants' safety, we excluded those at acute suicide risk and advised participants at each EMA prompt to contact clinical staff and use their safety plan when feeling distressed. As part of the follow‐up assessment, participants received up to €70 in cash. All study procedures received approval from the Ethical Review Board of the University of Ulm (197/23).

### 2.3 Measures

#### 2.3.1 Baseline Assessment

Participants completed a self‐report baseline assessment, including demographic information (e.g., age, sex at birth), the German version of the Impulsive Behavior Short Scale (eight items; α = 0.76; Kovaleva et al. 2012), the Perseverative Thinking Questionnaire (15 items; α = 0.91; Ehring et al. 2011), and a self‐translated version of the Suicidal Ideation Attributes Scale (SIDAS; 5 items; last 7 days; α = 0.75; van Spijker et al. 2014).

#### 2.3.2 EMA Items

At each prompt, participants rated their current level of passive and active SI on a 5‐point Likert scale (1 = not at all, 5 = extremely). Two items assessed passive SI (‘Life is not worth living for me’; ‘There are more reasons to die than to live for me’), and two items assessed active SI (‘I think about taking my life’; ‘I want to die’). We introduced each item with ‘At the moment,…’ to capture participants' momentary experiences. We created sum scores for both passive and active SI (range = 2–10) and computed an overall sum score (range = 4–20) across all SI items, developed and validated by Forkmann et al. (Forkmann et al. 2018).

At each prompt, participants also reported the activity they were engaged in (i.e., school, therapy, sport/PA, other leisure activity, resting/sleeping, eating/drinking, other activities) and who was with them at the time of responding to the prompt (i.e., alone, with other adolescents from the clinic, clinic staff/teachers, friends/acquaintances, family/relatives, someone else). The latter allowed multiple responses. See Table S1 for more details on EMA items.

### 2.4 Data Analysis

#### 2.4.1 Descriptive and Variability Statistics

We conducted all analyses in R (version 4.5.1). For the SI items, we computed descriptive statistics (mean, standard deviation [SD], range) using participant‐level weighting to account for unequal numbers of assessments across participants and calculated several variability measures. The intraclass correlation coefficient (ICC) quantifies the proportion of variance attributable to between‐person differences, with 1 − ICC representing within‐person variability (Eisele et al. 2021). The within‐person variance (WPV) quantifies the extent to which an individual's SI scores fluctuate around their own mean level over time (Jahng et al. 2008). The mean square of successive differences (MSSD) assesses temporal variability by measuring changes between successive observations, with higher scores indicating greater fluctuations (Jahng et al. 2008). Additionally, we calculated its root mean square of successive differences (RMSSD), which expresses variability in the original scale units. Based on previous research computing MSSD (Hallensleben et al. 2018b), we handled missing data by removing missing time points and shortening the time series. The probability of acute change (PAC) captures how often extreme shifts occur between successive measurements. This number is divided by the total number of possible successive comparisons, resulting in a value ranging from 0 to 1, with higher values indicating greater instability (Jahng et al. 2008). Whereas a previous study (Jacobucci et al. 2023) used a 5‐point Likert scale and defined 1‐ and 2‐point changes in active SI as extreme, we added another threshold representing 3‐point changes and refined the operational definition of extreme shifts to align with our broader sum scales. Accordingly, we defined extreme shifts as changes exceeding 4, 8, and 12 points for overall SI and 2, 4, and 6 points for passive and active SI, with thresholds termed PAC^low^, PAC^medium^, and PAC^high^, respectively. Additionally, we analyzed variability graphically, illustrating SI dynamics with individual plots and heat maps using the R‐package *ggplot*2 (Wickham 2016).

#### 2.4.2 Multilevel Analyses

To examine the influence of temporal and contextual factors on overall, passive, and active SI, we fitted multilevel models using the R package *lme4*. The first model (m1) captured the association between time of day and SI. Based on the time of prompt entry, we differentiated between morning (7:00–8:59 a.m.), late morning (9:00–11:59 a.m.), early afternoon (12:00–2:59 p.m.), afternoon (3:00–5:59 p.m.), and evening (6:00–9:00 p.m.), using evening as reference category. The second model (m2) examined the association between the day of the week and SI. Therefore, we differentiated between the weekdays (i.e., ‘Monday’, ‘Tuesday’, ‘Wednesday’, etc.) and used Monday as reference category. To account for weekday effects, we applied a three‐level model with day included as an additional level. The third model (m3) examined the association between activity and SI. Based on participants' reported activity at each prompt, we differentiated between ‘physical activity’ and non‐physical activities (school, therapy, other leisure activity, resting/sleeping, eating/drinking, other activities). Finally, to examine social context effects on SI (m4), we differentiated between moments where adolescents reported being alone and moments where they reported being together with others (other adolescents from the clinic, clinic staff/teachers, friends/acquaintances, family/relatives, someone else).

In line with previous research (Hallensleben et al. 2018a), we estimated all models using restricted maximum likelihood. To improve estimation accuracy, we included random slopes where permissible based on model complexity. All models included additional predictor variables to control for potential variables associated with missing values (i.e., age, sex, impulsivity, ruminative thinking, baseline SIDAS). We calculated *p*‐values using Satterthwaite approximation via *lmerTest*; *p*‐values < 0.05 were considered statistically significant.

## 3 Results

### 3.1 Sample Characteristics

The sample consisted of *N* = 51 participants aged 12–18 years (*M* = 15.70, SD = 1.44). The majority (80.4%) reported being assigned female sex at birth. At baseline, 72.5% (*n* = 37) reported one or more prior suicide attempts; the average SIDAS sum score was 22.02 (SD = 11.01; range 0–43, within a possible 0–50 range).

### 3.2 Characteristics of EMA Data

In total, 2485 assessments were distributed. All participants completed the study; two adolescents (ID12, ID26) participated for only 6 days due to early discharge. Based on the completed SI data, the overall adherence rate was 78.1% (range: 22.5%–100%). The final dataset comprised 1941 valid assessments. Table S2 provides repeated‐measures correlations (Bakdash and Marusich 2017), showing positive associations among the SI items and between the two SI subscales within individuals, suggesting that passive and active SI represent related but distinguishable aspects of SI.

### 3.3 Descriptive Statistics

Table 1 presents descriptive statistics (mean, SD, range) for overall, passive, and active SI. The mean for overall SI was 9.13, with scores ranging from 4.00 to 17.21. Passive SI showed a mean of 5.10, whereas the mean of active SI was slightly lower (*M* = 4.03). The observed ranges in passive and active SI were comparable, extending up to 8.67 and 8.54, respectively. Table 2 presents descriptive statistics of contextual factors. During the EMA phase, adolescents reported being engaged in PA in 4.5% of cases. Regarding the social context, being alone (35.8%) was the second most frequently reported social context.

**TABLE 1:** Descriptive statistics of suicidal ideation.

Constructs	Mean^a^	SD^a^	Range^a^
Overall suicidal ideation	9.13	3.30	4.00–17.21
Passive suicidal ideation	5.10	1.86	2.00–8.67
Active suicidal ideation	4.03	1.57	2.00–8.54

^a^ Analyzed based on sum scores (overall SI: 4–20; passive/active SI: 2–10).

**TABLE 2:** Descriptive statistics of contextual factors.

Activity	*n* (%)	Social context^a^	*n* (%)
School	124 (6.4%)	Alone	694 (35.8%)
Therapy	145 (7.5%)	Other adolescents from the clinic	795 (41.0%)
Physical activity	87 (4.5%)	Clinic staff/teachers	545 (28.1%)
Other leisure activity	452 (23.3%)	Friends/acquaintances	219 (11.3%)
Resting/sleeping	381 (19.6%)	Family/relatives	189 (9.7%)
Eating/drinking	281 (14.5%)	Someone else	59 (3.0%)
Other	471 (24.3%)		

^a^ Multiple responses allowed.

### 3.4 Variability of SI

Variability statistics (ICC, WPV, MSSD, RMSSD, PAC) are presented in Table 3. The ICC indicated that 58% of the variance in overall SI was attributable to between‐person variability, whereas 42% was due to within‐person variability (see Table S3 for further details on the ICCs of the individual SI items). Further, WPV indicated notable within‐person variability in SI (*M* = 7.51 overall SI; 2.40 passive SI; 2.10 active SI), along with substantial between‐person differences. While some participants showed little to no variability across measurement occasions, others exhibited pronounced fluctuations, with scores up to 7.80 for passive SI and 6.43 for active SI. The RMSSD scores further indicated that, on average, overall SI fluctuated by 2.85 points between measurement occasions, and by 1.71 and 1.47 points for passive and active SI, respectively. Additionally, under the PAC^low^ definition, such shifts occurred in 16% and 13% of passive and active SI measurements, respectively. When applying the PAC^medium^‐threshold, only 2% of measurements for both passive and active SI were classified as extreme shifts. Although the mean PAC^high^ scores for passive and active SI in the total sample were 0.00 (SD = 0.00), individual values reached up to 0.05 (*n* = 2) and 0.03 (*n* = 1), respectively, indicating that fluctuations > 6 points occurred in a few isolated cases.

**TABLE 3:** Variability statistics of suicidal ideation.

Constructs	ICC	WPV^a^	MSSD^a^	RMSSD^a^	PAC^low^ ^a^	PAC^medium^ ^a^	PAC^high^ ^a^
Overall suicidal ideation	0.58						
Mean (SD)		7.51 (5.52)	9.71 (7.39)	2.85 (1.28)	0.14 (0.14)	0.02 (0.03)	0.00 (0.00)
Range		0.00–20.38	0.00–27.88	0.00–5.28	0.00–0.46	0.00–0.11	0.00–0.03
Passive suicidal ideation	0.58						
Mean (SD)		2.40 (1.73)	3.40 (2.35)	1.71 (0.69)	0.16 (0.15)	0.02 (0.04)	0.00 (0.01)
Range		0.00–7.80	0.00–10.92	0.00–3.30	0.00–0.50	0.00–0.16	0.00–0.05
Active suicidal ideation	0.53						
Mean (SD)		2.10 (1.59)	2.78 (2.29)	1.47 (0.79)	0.13 (0.13)	0.02 (0.04)	0.00 (0.01)
Range		0.00–6.43	0.00–8.77	0.00–2.96	0.00–0.46	0.00–0.19	0.00–0.03

*Note:* PAC^low^, PAC^medium^, and PAC^high^ indicate extreme shifts (overall SI:4, 8, and 12; passive/active SI: 2,4, and 6).

Abbreviations: (R)MSSD = (root) mean square of successive differences, ICC = intraclass correlation coefficient, PAC = probability of acute change, WPV = within‐person variance.

^a^ Analyzed based on sum scores (overall SI: 4–20; passive/active SI: 2–10).

Figure 1 displays SI trajectories for two exemplary participants with low (ID55, ID45) and two with high (R)MSSD scores (ID15, ID47)—illustrating that some participants reported temporal stability in SI, whereas others showed high fluctuations. Figure S1 provides individual time series plots of all 51 participants, sorted by their overall SI (R)MSSD.

**FIGURE 1:**
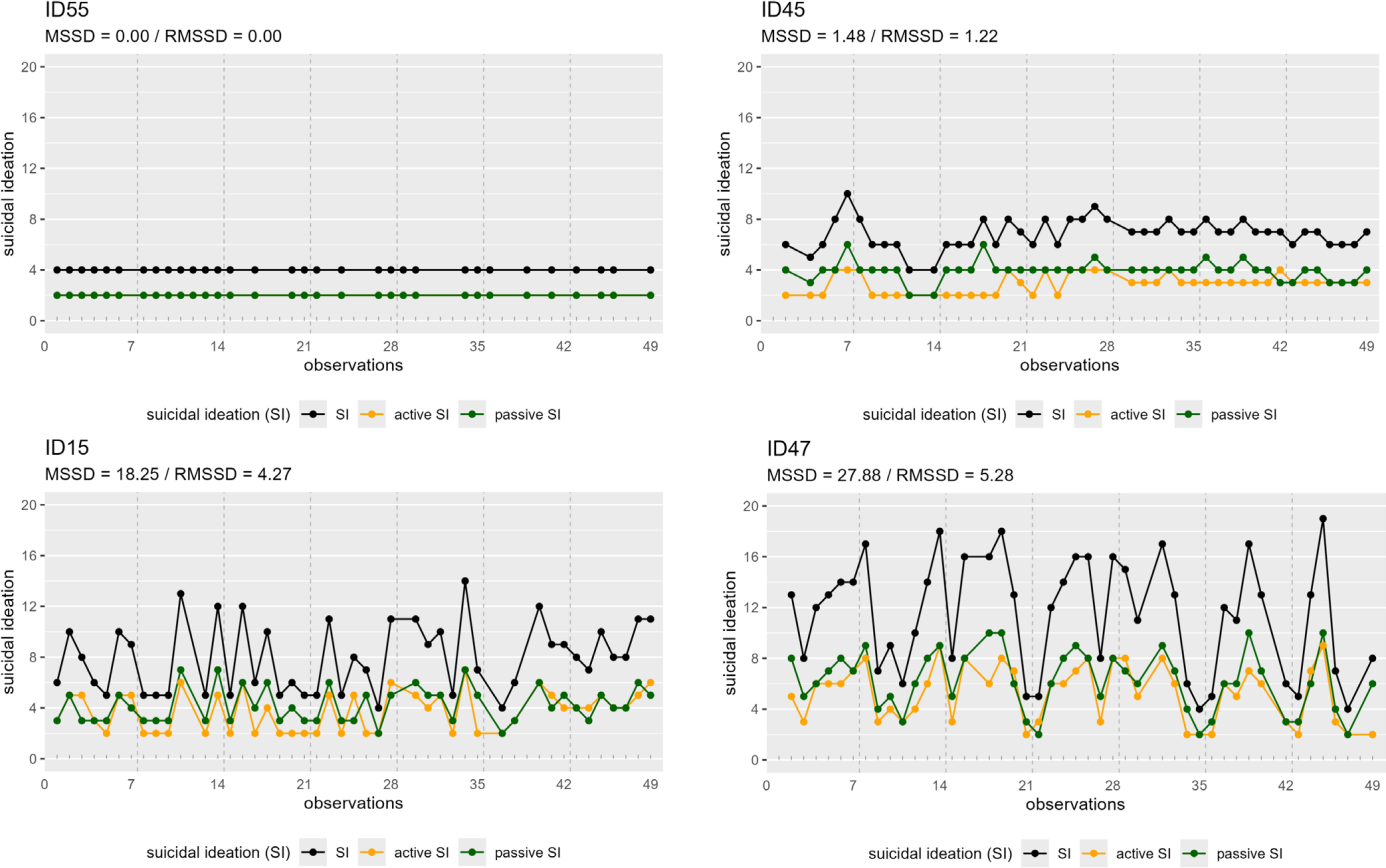
Exemplary time series plots. Trajectories for exemplary participants, showing overall SI (range 4–20), as well as passive and active SI separately (range 2–10), based on 7 daily prompts on 7 days. (R)MSSD = (root) mean square of successive differences.

Figure 2 presents a heat map of overall SI ratings across all observations and participants, with color changes within rows indicating within‐participant variability over time, while differences in patterns across rows illustrate variability between participants. Figure S2 illustrates heat maps of passive and active SI, highlighting that variability differs between passive and active SI, with higher variability observed in passive SI.

**FIGURE 2:**
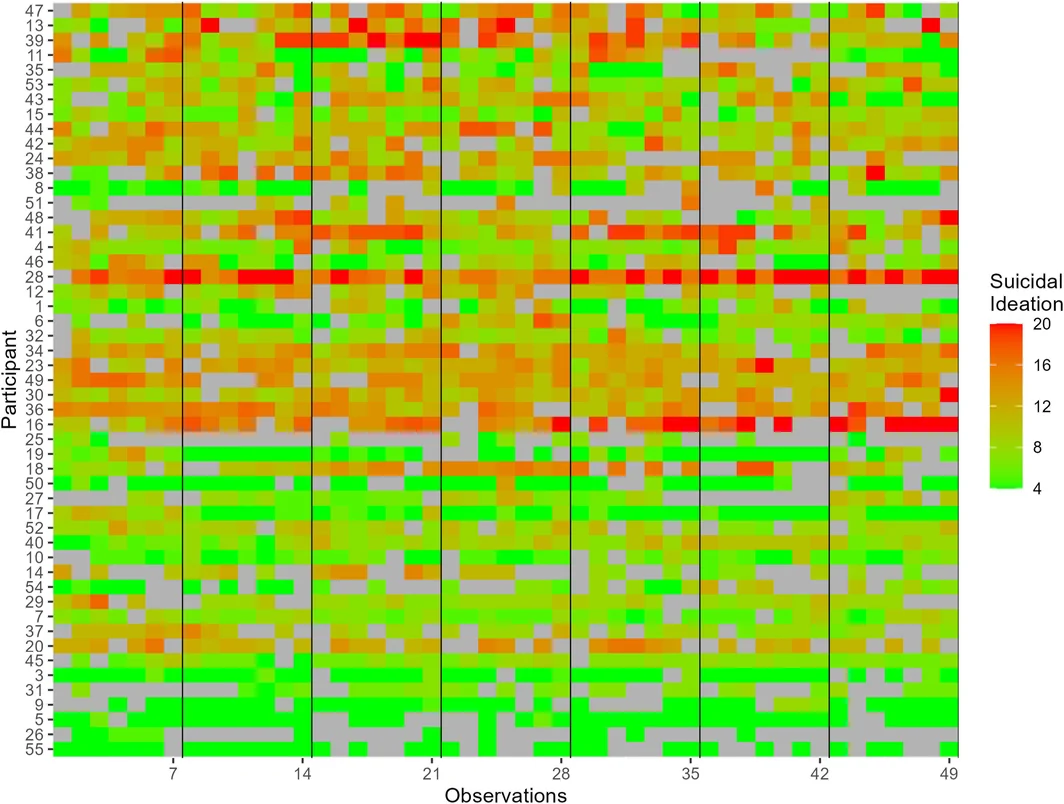
Heat map of overall SI. The ratings of current SI are color‐coded (green = low level; red = high level; gray = missing). Each row represents one participant, and each square corresponds to one rating. The x‐axis displays the 49 prompts (Days 1–7 separated by vertical lines); the y‐axis orders participants by their overall SI MSSD (higher at top, lower at bottom).

### 3.5 Temporal and Contextual Factors

Table 4 shows the effects of temporal and contextual factors on SI. In line with H1, adolescents reported significantly lower levels of overall SI in the morning and afternoon than in the evening. For active SI, participants reported lower levels during the day than in the evening, whereas no significant time‐of‐day effects were observed for passive SI. Regarding H2 (day‐of‐week effects), participants reported significantly lower overall and active SI on Thursdays compared to Mondays. Specifically, overall SI showed a decreasing trend toward the end of the workweek but did not reach statistical significance. Differences for all other days of the week were also non‐significant. As predicted in H3, adolescents reported significantly less passive SI in moments where they reported being engaged in PA than in moments where they were not engaged in PA. Contrary to H4, no significant differences in SI emerged between moments when adolescents were alone and when they were with others.

**TABLE 4:** Results of multilevel models: Association between temporal and contextual factors and suicidal ideation.

Constructs	Overall suicidal ideation			Passive suicidal ideation			Active suicidal ideation		
Predictors	*B*	SE	*p*	*B*	SE	*p*	*B*	SE	*p*
*Time of day*	(*m1.1* ^a^)			(*m1.2* ^a^)			(*m1.3* ^a^)		
Morning (7:00–08:59 a.m.)	−0.13	0.05	**0.017** *	−0.09	0.06	0.137	−0.17	0.06	**0.003** **
Late morning (9:00–11:59 a.m.)	−0.09	0.05	0.075	−0.06	0.06	0.313	−0.12	0.05	**0.022** *
Early afternoon (12:00–2:59 p.m.)	−0.08	0.05	0.069*	−0.05	0.05	0.291	−0.11	0.05	**0.021** *
Afternoon (3:00–5:59 p.m.)	−0.11	0.05	**0.030** *	−0.07	0.05	0.209	−0.14	0.05	**0.006** **
Evening (reference)	—	—	—	—	—	—	—	—	—
Marginal R^2^/Conditional R^2^	0.13/0.60			0.15/0.60			0.09/0.55		
*Day of the week*	(*m2.1* ^a^)			(*m2.2* ^a^)			(*m2.3* ^a^)		
Monday (reference)	—	—	—	—	—	—	—	—	—
Tuesday	−0.11	0.09	0.232	−0.13	0.10	0.179	−0.08	0.09	0.369
Wednesday	−0.09	0.09	0.313	−0.13	0.10	0.176	−0.05	0.09	0.581
Thursday	−0.19	0.09	**0.031** *	−0.16	0.10	0.087	−0.22	0.09	**0.019** *
Friday	−0.16	0.09	0.064	−0.16	0.10	0.103	−0.17	0.09	0.066
Saturday	−0.10	0.09	0.279	−0.03	0.10	0.771	−0.16	0.09	0.083
Sunday	−0.11	0.09	0.191	−0.07	0.10	0.450	−0.16	0.09	0.090
Marginal R^2^/Conditional R^2^	0.13/0.69			0.16/0.68			0.09/0.65		
*Activity*	(*m3.1*)			(*m3.2*)			(*m3.3* ^a^)		
Non‐physical activities (reference)	—	—	—	—	—	—	—	—	—
Physical activity	−0.15	0.08	0.064	−0.21	0.09	**0.025** *	−0.09	0.08	0.271
Marginal R^2^/Conditional R^2^	0.13/0.60			0.15/0.60			0.09/0.55		
*Social context*	*(m4.1)*			*(m4.2)*			*(m4.3)*		
Being together with others (reference)	—	—	—	—	—	—	—	—	—
Alone	0.06	0.04	0.136	0.08	0.04	0.060	0.04	0.05	0.426
Marginal R^2^/Conditional R^2^	0.13/0.60			0.15/0.60			0.09/0.55		

*Note:* m1 + m3 + m4: Assessments (Level 1) *N* = 1.941, Participants (Level 2) *N* = 51. m2: Assessments (Level 1) *N* = 1.941, Day of the week (Level 2) *N* = 351, Participants (Level 3) *N* = 51.

* *p* < 0.05.

** *p* < 0.01.

*** *p* < 0.001. Bold indicates significant values at the *p* < 0.05 level.

^a^ Model was fitted with fixed slopes.

As sensitivity analyses, we conducted two‐part mixed‐effects models to examine whether the high proportion of assessments with no reported SI affected the primary findings. No SI was reported in 18.8% of assessments for overall SI, 19.8% for passive SI, and 34% for active SI. The two‐part models comprised a logistic mixed‐effects occurrence component (i.e., scores > 4 for overall SI and > 2 for passive and active SI) and a mixed‐effects severity component restricted to assessments in which SI was present. Both components were fitted using the *lme4* package in R, retaining the fixed‐ and random‐effects structure of the primary analyses wherever feasible. Results of the two‐part mixed‐effect models conducted as sensitivity analyses are presented in Tables S4–S7. Overall, significant associations pertained mainly to the severity rather than the occurrence component. Additional associations emerged across temporal and contextual factors; for instance, several day‐of‐week contrasts suggested lower SI severity relative to Monday.

### 3.6 Supplementary Analyses

We tested the influence of different activities (school, therapy, other leisure activities, resting/sleeping, eating/drinking) and the presence of specific persons (other adolescents from the clinic, clinic staff/teachers, friends/acquaintances, family/relatives) on momentary SI (see Tables S8 and S9). The results indicated that adolescents reported significantly lower levels of SI in moments where they were being engaged in other leisure activities than in moments where they were not engaged in other leisure activities. By contrast, adolescents reported significantly higher levels of SI in moments where they reported being engaged in any ‘other activities’; although the specific types of activities included in this category remain unclear. Regarding the social context, adolescents reported significantly lower levels of overall and active SI in moments where they were with friends/acquaintances compared to moments when they were not. All other analyses were not statistically significant.

## 4 Discussion

This study examined the variability and the influence of temporal and contextual factors on SI among inpatient adolescents. The main findings are twofold: First, SI among adolescents showed substantial variability in overall, passive, and active SI. Second, the multilevel models showed significant effects of time of day, day of the week, and PA on SI.

### 4.1 Variability of SI

In our study, 42% of the variance in overall SI was due to within‐person variability, which is comparable to findings from previous EMA studies: Czyz et al. (Czyz et al. 2019; 4 weeks with 1 daily prompt) reported within‐person variability of 43%–47% across SI frequency, duration, and urge, whereas Andrewes et al. (Andrewes et al. 2024; 7 days with six daily prompts) reported 54% within‐person variability among 15‐ to 25‐year‐olds with BPD. Similarly, previous EMA studies among young adults with recent or lifetime SI reported within‐person variability of 51% and 56%, respectively (Oakey‐Frost et al. 2023; Gerner et al. 2023). In contrast, EMA studies among adults have shown lower proportions of within‐person variability in SI (25%–33%; Forkmann et al. 2018; Kleiman et al. 2017; Silva et al. 2022), suggesting that within‐person variability constitutes a larger proportion of the total variance in younger populations. Regarding passive and active SI, an EMA study among adults reported within‐person variability of 25% and 36% (Hallensleben et al. 2018a), whereas we found higher values of 42% and 47%, respectively. Additionally, the wide WPV and (R)MSSD ranges indicate substantial between‐participant differences. Taken together, previous and present findings suggest that SI among adolescents—both passive and active—show substantial within‐person variability and between‐person differences.

Although the MSSD is not comparable across studies using different items, it allows comparison with one previous EMA study using the same SI items among inpatient adults (Hallensleben et al. 2018a, Hallensleben et al. 2018b; 6 days with 10 daily prompts). Compared to this study, our adolescent sample showed higher average MSSD values for overall SI (9.71 vs. 5.1), passive SI (3.40 vs. 1.95), and active SI (2.78 vs. 1.39). This comparison further supports the suggested greater within‐person variability in SI among adolescents than adults. The higher MSSD we observed for passive SI compared to active SI should be interpreted cautiously because passive SI was reported more frequently, so its higher variability likely reflects reporting frequency rather than substantial differences in instability.

The PAC^low^ value for overall SI indicated that 14% of successive SI assessments exceeded an extreme 4‐point shift, suggesting notable instability in SI among adolescents. In our adolescent sample, 13% of measurements exceeded the PAC^low^‐threshold in active SI, which is comparable to findings by Jacobucci et al. (Jacobucci et al. 2023), who assessed active SI among young adults (mean age = 25.88) using a single item. They reported only 8.7% of measurements exceeding a similar PAC^low^‐threshold, which suggests greater instability in our adolescent sample at a low threshold. When applying a higher threshold (PAC^medium^), the results were comparable (2% vs. 2.7%), suggesting that large fluctuations are rare. Observed PAC^high^ values showed that extreme shifts were unlikely. In general, PAC ranges indicated substantial interindividual variability: some participants showed no instability, others pronounced fluctuations, highlighting sample heterogeneity. These results suggest that the observed PAC values align with other variability measures; however, a comparison with previous studies is not possible due to a lack of research applying PAC to SI.

### 4.2 Temporal and Contextual Factors

The results on the relationship between time of day and SI showed that active SI occurred most frequently toward the evening, which is consistent with findings from studies among adults (e.g., Kim et al. 2025). A retrospective study of inpatient adolescents who attempted suicide found that most attempts occurred between evening and midnight (Akkaya‐Kalayci et al. 2017), suggesting that SI may also be heightened during this time. This pattern has been attributed to increased opportunities for self‐reflection and the absence of occupation or structure. Such periods may be associated with the emergence of negative thoughts, which might be further amplified in inpatient settings where mornings and evenings are typically less structured than daytime hours (e.g., school, therapy sessions). Contrary to our hypothesis and previous findings (Freichel and O'Shea 2023; Hatton et al. 2024; Kim et al. 2019), we observed significantly increased SI on Mondays only compared to Thursdays, while effects for the other days were non‐significant. These results may be explained, in part, by the inpatient setting, where the typical weekly structure and external stressors (e.g., returning to school or work) are largely absent. Similarly, regarding the influence of weekends on SI, our findings showed no significantly lower levels of SI on weekend days, which may be explained by trial periods outside the clinic, during which inpatients are allowed to leave the clinic for temporary everyday‐life exposure and are therefore no longer in the structured and potentially stabilizing inpatient setting. In contrast, Lee et al. (Lee et al. 2024) observed a reduced risk of SI on weekends in Europe.

Adolescents reported significantly reduced passive, but not active SI, in moments where they reported being engaged in PA, compared to moments where they were involved in other activities. One possible explanation may be that PA reduces passive SI by promoting well‐being and stress relief, which is not sufficient for severe forms of SI. This aligns with evidence linking PA to better mental health outcomes in general, in both adolescent and young adult populations (McMahon et al. 2017; Heß and Weiss 2024; Grasdalsmoen et al. 2020). Contrary to our expectations, the hypothesized protective presence of others was not supported by our results. Previous studies have suggested that social support may buffer against SI (Heß and Weiss 2024; Brown and Scott 2025); however, we merely assessed the presence of others—not the perceived quality of those interactions.

### 4.3 Strengths and Limitations

Key strengths of this study include the use of validated SI EMA items in a high‐risk inpatient adolescent sample, and the differentiation between passive and active SI. Despite excluding adolescents with acute suicide risk, baseline SIDAS scores were high, indicating a high‐risk sample. Furthermore, we included more daily prompts than previous adolescent studies to better capture variability. However, because data collection ended at 9:00 p.m., SI levels later at night—potentially higher than those observed—could not be captured. During the follow‐up assessment, some participants mentioned increased SI after 9:00 p.m., highlighting the need for late‐night data collection. Because no additional information on adolescents' daily lives was collected, the influence of other sources of daily stress like challenging family conversations (Novotny et al. 2025) could not be considered. Further, we did not assess the category ‘other activities’ in detail; therefore, the specific activities associated with higher SI remained unclear. Moreover, the inpatient setting limits generalizability, as inpatients often experience more severe and chronic mental health issues (Kipoulas et al. 2020; Haavisto et al. 2003) and their routines may differ from everyday life. Finally, this structured, relatively protected setting and its relatively homogeneous social and environmental context may reduce exposure to everyday stressors and restrict contextual variability, which may, in turn, attenuate SI variability and limit the detection of context‐related differences.

## 5 Conclusion

The present study examined variability of overall, passive and active SI and the influence of temporal and contextual factors on SI, thereby offering valuable insights into the SI dynamics among adolescents. The observed substantial short‐term variability highlights the need for continuous and context‐sensitive assessment of SI to detect periods of elevated risk (e.g., during the evening). Such assessments can enable timely and tailored interventions to reduce suicide risk among adolescents. Building on the observed interindividual differences in SI variability, future research should clarify the clinical relevance of distinct patterns of SI variability. This may improve understanding of individual SI trajectories and inform the development of tailored suicide prevention strategies for adolescents.

## Author Contributions

**Tamara Großmann:** conceptualization, writing – original draft, writing – review and editing, visualization, methodology, formal analysis, data curation, investigation. **Matthias Lühr:** conceptualization, writing – review and editing, methodology, formal analysis, data curation, investigation. **Nadine Bayer:** project administration, conceptualization, methodology. **Jörg M. Fegert:** resources, writing – review and editing. **Verena Gindele:** resources, writing – review and editing. **Sophie Köster:** resources, writing – review and editing. **Sabine Müller:** resources, writing – review and editing. **Miriam Rassenhofer:** supervision, writing – review and editing, resources. **Christian Rauschenberg:** writing – review and editing, methodology. **Ulrich Reinighaus:** writing – review and editing, methodology. **Theresa Schockenhoff:** resources, writing – review and editing. **Ulrike M. E. Schulze:** resources, writing – review and editing. **Heike Speth:** resources, writing – review and editing. **Nathalie Oexle:** conceptualization, supervision, writing – review and editing, methodology, formal analysis, funding acquisition.

## Funding

This research was funded by the German Federal Ministry of Research, Technology and Space (01GY2104).

## Ethics Statement

The study followed the Declaration of Helsinki and received ethical approval from the Ethical Review Board of the University of Ulm (197/23).

## Consent

Written informed consent was obtained from all participants and, for minors, their legal guardians.

## Conflicts of Interest

The authors declare no conflicts of interest.

## Supporting information


**Table S1:** EMA items.

**Table S2:** Repeated‐measures correlations among suicidal ideation items and subscales.

**Table S3:** Intraclass correlation for suicidal ideation items.

**Table S4:** Two‐part sensitivity analyses for time of day.
**Table S5:** Two‐part sensitivity analyses for day of the week.
**Table S6:** Two‐part sensitivity analyses for activity.
**Table S7:** Two‐part sensitivity analyses for social context.

**Table S8:** Results of multilevel models for activities.

**Table S9:** Results of multilevel models for social context.

**Figure S1:** Time series plots.

**Figure S2:** Heat maps.

## Data Availability

The data from the EMA study are not publicly available due to privacy or ethical restrictions. The R syntax that supports the findings of this study is available upon request from the corresponding author.
