# Supplementary material for: Variability and Influencing Factors of Suicidal Ideation in Adolescents—An Ecological Momentary Assessment Study
Source: Suicide Life Threat Behav. 2026 Nov 8;56(6):e70149. doi: 10.1111/sltb.70149 (PMC13745968; doi:10.1111/sltb.70149)
Supplement: Supplementary file 1 [file SLTB-56-0-s001.docx]

**Table S4**

*Two-Part Sensitivity Analyses for Time of Day*

|  | Original Model | | | Two-Part Sensitivity Model | | | | | |  |
| --- | --- | --- | --- | --- | --- | --- | --- | --- | --- | --- |
|  |  | | | Occurrence Component | | | Severity Component | | |  |
|  | *B* | SE | p | OR | 95% CI | p | *B* | SE | p |  |
| *Overall Suicidal Ideation* |  |  |  |  |  |  |  |  |  |  |
| Morning (7:00–08:59 a.m.) | -0.13 | 0.05 | **0.017*** | 0.76 | 0.50–1.17 | 0.213 | -0.18 | 0.07 | **.010*** |  |
| Late morning (9:00–11:59 a.m.) | -0.09 | 0.05 | 0.075 | 0.67 | 0.46–0.98 | **0.040*** | -0.11 | 0.06 | .082 |  |
| Early afternoon (12:00–2:59 p.m.) | -0.08 | 0.05 | 0.069 | 0.68 | 0.48–0.97 | **0.033*** | -0.04 | 0.06 | .470 |  |
| Afternoon (3:00–5:59 p.m.) | -0.11 | 0.05 | **0.030*** | 0.80 | 0.55–1.17 | 0.257 | -0.16 | 0.06 | **.007**** |  |
| Evening (reference) | - | - | - | - | - | - | - | - | - |  |
|  |  |  |  |  |  |  |  |  |  |  |
| *Passive Suicidal Ideation* |  |  |  |  |  |  |  |  |  |  |
| Morning (7:00–08:59 a.m.) | -0.09 | 0.06 | 0.137 | 1.12 | 0.62–2.01 | 0.706 | -0.13 | 0.07 | 0.080 |  |
| Late morning (9:00–11:59 a.m.) | -0.06 | 0.06 | 0.313 | 1.03 | 0.61–1.74 | 0.907 | -0.06 | 0.07 | 0.325 |  |
| Early afternoon (12:00–2:59 p.m.) | -0.05 | 0.05 | 0.291 | 0.96 | 0.59–1.55 | 0.864 | -0.00 | 0.06 | 0.993 |  |
| Afternoon (3:00–5:59 p.m.) | -0.07 | 0.05 | 0.209 | 0.77 | 0.47–1.27 | 0.311 | -0.09 | 0.06 | 0.183 |  |
| Evening (reference) | - | - | - | - | - | - | - | - | - |  |
|  |  |  |  |  |  |  |  |  |  |  |
| *Active Suicidal Ideation* |  |  |  |  |  |  |  |  |  |  |
| Morning (7:00–08:59 a.m.) | -0.17 | 0.06 | **0.003**** | 0.79 | 0.50–1.27 | 0.333 | -0.24 | 0.07 | **0.001**** |  |
| Late morning (9:00–11:59 a.m.) | -0.12 | 0.05 | **0.022*** | 1.02 | 0.67–1.56 | 0.926 | -0.22 | 0.06 | **< .001***** |  |
| Early afternoon (12:00–2:59 p.m.) | -0.11 | 0.05 | **0.021*** | 0.90 | 0.61–1.32 | 0.583 | -0.17 | 0.06 | **0.005**** |  |
| Afternoon (3:00–5:59 p.m.) | -0.14 | 0.05 | **0.006**** | 1.09 | 0.72–1.65 | 0.699 | -0.26 | 0.06 | **< .001***** |  |
| Evening (reference) | - | - | - | - | - | - | - | - | - |  |

*Note.* B = unstandardized regression coefficient; SE = standard error; OR = odds ratio; CI = confidence interval. The occurrence component models whether SI is present, whereas the severity component models the SI severity conditional on SI being present.

*p < .05, **p < .01, ***p < .001. Bold indicates significant values at the p < .05 level.

**Table S5**

*Two-Part Sensitivity Analyses for Day of the Week*

|  | Original Model | | |  | Two-Part Sensitivity Model | | | | | |  |
| --- | --- | --- | --- | --- | --- | --- | --- | --- | --- | --- | --- |
|  |  | | |  | Occurrence Component | | | Severity Component | | |  |
|  | *B* | SE | p | OR | | 95% CI | p | *B* | SE | p |  |
| *Overall Suicidal Ideation* |  |  |  |  | |  |  |  |  |  |  |
| Monday (reference) | - | - | - | - | | - | - | - | - | - |  |
| Tuesday | -0.11 | 0.09 | 0.232 | 0.82 | | 0.41–1.65 | 0.577 | -0.21 | 0.10 | **0.032*** |  |
| Wednesday | -0.09 | 0.09 | 0.313 | 0.90 | | 0.45–1.79 | 0.757 | -0.14 | 0.10 | 0.150 |  |
| Thursday | -0.19 | 0.09 | **0.031*** | 0.80 | | 0.39–1.62 | 0.533 | -0.26 | 0.10 | **0.008**** |  |
| Friday | -0.16 | 0.09 | 0.064 | 0.73 | | 0.36–1.48 | 0.386 | -0.22 | 0.10 | **0.027*** |  |
| Saturday | -0.10 | 0.09 | 0.279 | 1.08 | | 0.54–2.14 | 0.828 | -0.20 | 0.10 | **0.032*** |  |
| Sunday | -0.11 | 0.09 | 0.191 | 0.99 | | 0.49–1.98 | 0.975 | -0.24 | 0.10 | **0.013*** |  |
|  |  |  |  |  | |  |  |  |  |  |  |
| *Passive Suicidal Ideation* |  |  |  |  | |  |  |  |  |  |  |
| Monday (reference) | - | - | - | - | | - | - | - | - | - |  |
| Tuesday | -0.13 | 0.10 | 0.179 | 0.96 | | 0.40–2.26 | 0.917 | -0.21 | 0.10 | **0.030*** |  |
| Wednesday | -0.13 | 0.10 | 0.176 | 0.96 | | 0.41–2.26 | 0.922 | -0.17 | 0.10 | 0.078 |  |
| Thursday | -0.16 | 0.10 | 0.087 | 0.73 | | 0.32–1.70 | 0.469 | -0.20 | 0.10 | **0.042*** |  |
| Friday | -0.16 | 0.10 | 0.103 | 0.76 | | 0.33–1.78 | 0.533 | -0.20 | 0.10 | **0.045*** |  |
| Saturday | -0.03 | 0.10 | 0.771 | 1.13 | | 0.47–2.72 | 0.785 | -0.13 | 0.09 | 0.182 |  |
| Sunday | -0.07 | 0.10 | 0.450 | 1.83 | | 0.76–4.40 | 0.178 | -0.17 | 0.10 | 0.070 |  |
|  |  |  |  |  | |  |  |  |  |  |  |
| *Active Suicidal Ideation* |  |  |  |  | |  |  |  |  |  |  |
| Monday (reference) | - | - | - | - | |  | - | - | - | - |  |
| Tuesday | -0.08 | 0.09 | 0.369 | 1.86 | | 0.79–4.39 | 0.155 | -0.21 | 0.12 | 0.082 |  |
| Wednesday | -0.05 | 0.09 | 0.581 | 0.83 | | 0.36–1.92 | 0.670 | -0.11 | 0.12 | 0.353 |  |
| Thursday | -0.22 | 0.09 | **0.019*** | 0.94 | | 0.40–2.18 | 0.882 | -0.33 | 0.12 | **0.006**** |  |
| Friday | -0.17 | 0.09 | 0.066 | 0.86 | | 0.37–1.98 | 0.720 | -0.24 | 0.12 | **0.044*** |  |
| Saturday | -0.16 | 0.09 | 0.083 | 0.94 | | 0.40–2.18 | 0.882 | -0.28 | 0.12 | **0.017*** |  |
| Sunday | -0.16 | 0.09 | 0.090 | 1.32 | | 0.57–3.07 | 0.511 | -0.30 | 0.12 | **0.010*** |  |

*Note.* B = unstandardized regression coefficient; SE = standard error; OR = odds ratio; CI = confidence interval. The occurrence component models whether SI is present, whereas the severity component models the SI severity conditional on SI being present.

*p < .05, **p < .01, ***p < .001. Bold indicates significant values at the p < .05 level.

**Table S6**

*Two-Part Sensitivity Analyses for Activity*

|  | Original Model | | |  | Two-Part Sensitivity Model | | | | | |  |
| --- | --- | --- | --- | --- | --- | --- | --- | --- | --- | --- | --- |
|  |  | | |  | Occurrence Component | | | Severity Component | | |  |
|  | *B* | SE | p | OR | | 95% CI | p | *B* | SE | p |  |
| *Overall Suicidal Ideation* |  |  |  |  |  |  |  |  |  |  |  |
| Non-physical activities (reference) | - | - | - | - | | - | - | - | - | - |  |
| Physical activity | -0.15 | 0.08 | 0.064 | 0.70 | | 0.40–1.22 | 0.204 | -0.09 | 0.11 | 0.376 |  |
|  |  |  |  |  | |  |  |  |  |  |  |
| *Passive Suicidal Ideation* |  |  |  |  | |  |  |  |  |  |  |
| Non-physical activities (reference) | - | - | - | - | | - | - | - | - | - |  |
| Physical activity | -0.21 | 0.09 | **0.025*** | 0.68 | | 0.26–1.79 | 0.436 | -0.23 | 0.09 | **0.014*** |  |
|  |  |  |  |  | |  |  |  |  |  |  |
| *Active Suicidal Ideation* |  |  |  |  | |  |  |  |  |  |  |
| Non-physical activities (reference) | - | - | - | - | | - | - | - | - | - |  |
| Physical activity | -0.09 | 0.08 | 0.271 | 0.92 | | 0.47–1.81 | 0.814 | -0.08 | 0.13 | 0.545 |  |

*Note.* B = unstandardized regression coefficient; SE = standard error; OR = odds ratio; CI = confidence interval. The occurrence component models whether SI is present, whereas the severity component models the SI severity conditional on SI being present.

*p < .05, **p < .01, ***p < .001. Bold indicates significant values at the p < .05 level.

**Table S7**

*Two-Part Sensitivity Analyses for Social Context*

|  | Original Model | | |  | Two-Part Sensitivity Model | | | | | |  |
| --- | --- | --- | --- | --- | --- | --- | --- | --- | --- | --- | --- |
|  |  | | |  | Occurrence Component | | | Severity Component | | |  |
|  | *B* | SE | p | OR | | 95% CI | p | *B* | SE | p |  |
| *Overall Suicidal Ideation* |  |  |  |  |  |  |  |  |  |  |  |
| Being together with others (reference) | - | - | - | - | | - | - | - | - | - |  |
| Alone | 0.06 | 0.04 | 0.136 | 1.07 | | 0.74–1.54 | 0.731 | 0.08 | 0.04 | 0.065 |  |
|  |  |  |  |  | |  |  |  |  |  |  |
| *Passive Suicidal Ideation* |  |  |  |  | |  |  |  |  |  |  |
| Being together with others (reference) | - | - | - | - | | - | - | - | - | - |  |
| Alone | 0.08 | 0.04 | 0.060 | 1.28 | | 0.89–1.83 | 0.184 | 0.10 | 0.04 | **0.024*** |  |
|  |  |  |  |  | |  |  |  |  |  |  |
| *Active Suicidal Ideation* |  |  |  |  | |  |  |  |  |  |  |
| Being together with others (reference) | - | - | - | - | | - | - | - | - | - |  |
| Alone | 0.04 | 0.05 | 0.426 | 1.08 | | 0.81–1.45 | 0.595 | 0.06 | 0.06 | 0.260 |  |

*Note.* B = unstandardized regression coefficient; SE = standard error; OR = odds ratio; CI = confidence interval. The occurrence component models whether SI is present, whereas the severity component models the SI severity conditional on SI being present.

*p < .05, **p < .01, ***p < .001. Bold indicates significant values at the p < .05 level.
