# Supplementary material for: Variability and Influencing Factors of Suicidal Ideation in Adolescents—An Ecological Momentary Assessment Study
Source: Suicide Life Threat Behav. 2026 Nov 8;56(6):e70149. doi: 10.1111/sltb.70149 (PMC13745968; doi:10.1111/sltb.70149)
Supplement: Supplementary file 2 [file SLTB-56-0-s002.docx]

**Table S9**

*Results of Multilevel Models for Social Context*

| Constructs | Overall suicidal ideation | | | Passive suicidal ideation | | | Active suicidal ideation | | |
| --- | --- | --- | --- | --- | --- | --- | --- | --- | --- |
| Predictors | *B* | SE | p | *B* | SE | p | *B* | SE | p |
| *Other adolescents from the clinic* | | | |  |  |  |  |  |  |
| Adolescents | -0.00 | 0.05 | 0.947 | -0.03 | 0.05 | 0.523 | 0.03 | 0.05 | 0.618 |
| Marginal R² / Conditional R² | 0.12/0.60 |  |  | 0.14/0.60 |  |  | 0.08/0.56 |  |  |
|  |  |  |  |  |  |  |  |  |  |
| *Clinic staff/teachers* | | | |  |  |  |  |  |  |
| Staff/teachers | -0.02 | 0.09 | 0.828 | -0.09 | 0.10 | 0.386 | 0.06 | 0.08 | 0.464 |
| Marginal R² / Conditional R² | 0.14/0.61 |  |  | 0.16/0.61 |  |  | 0.10/0.55 |  |  |
|  |  |  |  |  |  |  |  |  |  |
| *Friends/acquaintances* | | | |  |  |  |  |  |  |
| Friends/ acquaintances | -0.17 | 0.08 | **0.036*** | -0.12 | 0.10 | 0.236 | -0.19 | 0.07 | **0.006****^a^ |
| Marginal R² / Conditional R² | 0.12/0.60 |  |  | 0.15/0.60 |  |  | 0.09/0.55 |  |  |
|  |  |  |  |  |  |  |  |  |  |
| *Family/relatives* | | | |  |  |  |  |  |  |
| Family/relatives | -0.01 | 0.07 | 0.838 | 0.04 | 0.08 | 0.594 | -0.07 | 0.07 | 0.337 |
| Marginal R² / Conditional R² | 0.14/0.60 |  |  | 0.17/0.61 |  |  | 0.08/0.54 |  |  |

*Note.* Assessments (level 1) N = 1.941, Participants (level 2) N = 51.

*p < .05, **p < .01, ***p < .001. Bold indicates significant values at the p < 0.05 level.

^a^ Model failed to converge with random slopes, therefore this model was fitted with fixed slopes.
