# Supplementary material for: Variability and Influencing Factors of Suicidal Ideation in Adolescents—An Ecological Momentary Assessment Study
Source: Suicide Life Threat Behav. 2026 Nov 8;56(6):e70149. doi: 10.1111/sltb.70149 (PMC13745968; doi:10.1111/sltb.70149)
Supplement: Supplementary file 3 [file SLTB-56-0-s003.docx]

**Table S8**

*Results of Multilevel Models for Activities*

| Constructs | Overall suicidal ideation | | | Passive suicidal ideation | | | Active suicidal ideation | | |
| --- | --- | --- | --- | --- | --- | --- | --- | --- | --- |
| Predictors | *B* | SE | p | *B* | SE | p | *B* | SE | p |
| *School* | | | |  |  |  |  |  |  |
| School | 0.05 | 0.09 | 0.599 | 0.01 | 0.09 | 0.943 | 0.09 | 0.10 | 0.388 |
| Marginal R² / Conditional R² | 0.13/0.60 |  |  | 0.15/0.60 |  |  | 0.09/0.55 |  |  |
|  |  |  |  |  |  |  |  |  |  |
| *Therapy* ^a^ | | | |  |  |  |  |  |  |
| Therapy | 0.01 | 0.06 | 0.852 | -0.04 | 0.07 | 0.576 | 0.06 | 0.07 | 0.344 |
| Marginal R² / Conditional R² | 0.13/0.60 |  |  | 0.15/0.60 |  |  | 0.09/0.55 |  |  |
|  |  |  |  |  |  |  |  |  |  |
| *Other leisure activities* | | | |  |  |  |  |  |  |
| Leisure activities | -0.15 | 0.05 | **0.003**** | -0.17 | 0.06 | **0.006**** | -0.14 | 0.05 | **0.003**** |
| Marginal R² / Conditional R² | 0.14/0.61 |  |  | 0.14/0.61 |  |  | 0.08/0.55 |  |  |
|  |  |  |  |  |  |  |  |  |  |
| *Resting/Sleeping* | | | |  |  |  |  |  |  |
| Resting/Sleeping | 0.02 | 0.05 | 0.680 | 0.08 | 0.05 | 0.139 | -0.04 | 0.05 | 0.449 |
| Marginal R² / Conditional R² | 0.13/0.60 |  |  | 0.15/0.60 |  |  | 0.09/0.55 |  |  |
|  |  |  |  |  |  |  |  |  |  |
| *Eating/Drinking ^a^* | | | |  |  |  |  |  |  |
| Eating/Drinking | -0.01 | 0.05 | 0.901 | 0.00 | 0.05 | 0.975 | -0.01 | 0.05 | 0.788 |
| Marginal R² / Conditional R² | 0.13/0.60 |  |  | 0.15/0.60 |  |  | 0.09/0.55 |  |  |
|  |  |  |  |  |  |  |  |  |  |
| *Other activity* |  |  |  |  |  |  |  |  |  |
| Other activity | 0.16 | 0.04 | **<.001***** | 0.16 | 0.05 | **<.001***** | 0.16 | 0.05 | **<.001***** |
| Marginal R² / Conditional R² | 0.14/0.61 |  |  | 0.17/0.61 |  |  | 0.09/0.55 |  |  |

*Note.* Assessments (level 1) N = 1.941, Participants (level 2) N = 51.

*p < .05, **p < .01, ***p < .001. Bold indicates significant values at the p < 0.05 level.

^a^ Model failed to converge with random slopes, therefore this model was fitted with fixed slopes.
