# Supplementary material for: Variability and Influencing Factors of Suicidal Ideation in Adolescents—An Ecological Momentary Assessment Study
Source: Suicide Life Threat Behav. 2026 Nov 8;56(6):e70149. doi: 10.1111/sltb.70149 (PMC13745968; doi:10.1111/sltb.70149)
Supplement: Supplementary file 4 [file SLTB-56-0-s004.docx]

**Table S1**

***EMA items***

| Item | Scale | Construct | Source |
| --- | --- | --- | --- |
| At the moment… |  |  |  |
| 1. I feel useless. | 1-5 | Perceived burdensomeness | Forkmann et al. 2018 |
| 2. I feel like a burden for others. | 1-5 | Perceived burdensomeness | Forkmann et al. 2018 |
| 3. I feel lonely. | 1-5 | Thwarted belongingness | Forkmann et al. 2018 |
| 4. I feel like I do not belong. | 1-5 | Thwarted belongingness | Forkmann et al. 2018 |
| 5. I feel cheerful. | 1-5 | Positive affect | Forkmann et al. 2018 |
| 6. I feel happy. | 1-5 | Positive affect | Forkmann et al. 2018 |
| 7. I feel like a winner. | 1-5 (reverse coded) | Defeat | Self-developed |
| 8. I succeed at everything | 1-5 (reverse coded) | Defeat | Self-developed |
| 9. I see an escape from my troubles. | 1-5 (reverse coded) | Entrapment | Self-developed |
| 10. I feel free. | 1-5 (reverse coded) | Entrapment | Self-developed |
| 11. Life is not worth living for me. | 1-5 | Passive suicidal ideation | Forkmann et al. 2018 |
| 12. There are more reasons to die than to live for me. | 1-5 | Passive suicidal ideation | Forkmann et al. 2018 |
| 13. I want to die. | 1-5 | Active suicidal ideation | Forkmann et al. 2018 |
| 14. I think about taking my life. | 1-5 | Active suicidal ideation | Forkmann et al. 2018 |
| 15. What are you doing? | - school - therapy - sport/physical activity - other leisure activity - resting/ sleeping - eating/ drinking - other | Context: activity | Influenced by Forkmann et al. 2018 |
| 16. Who is with you? | - I am alone. - other adolescents from the clinic - clinical staff/ teachers - friends/ acquaintances - family/ relatives - someone else | Context: social context | Influenced by Forkmann et al. 2018 |
