# Supplementary material for: Variability and Influencing Factors of Suicidal Ideation in Adolescents—An Ecological Momentary Assessment Study
Source: Suicide Life Threat Behav. 2026 Nov 8;56(6):e70149. doi: 10.1111/sltb.70149 (PMC13745968; doi:10.1111/sltb.70149)
Supplement: Supplementary file 5 [file SLTB-56-0-s005.docx]

**Figure S1**

*Time Series Plots*

*Note.* Trajectories for all participants (N = 51), sorted by their (R)MSSD-score for overall SI. Plots show overall SI (sum score of 4 SI items; range 4–20), as well as passive and active SI separately, based on 7 prompts per day over 7 consecutive days. (R)MSSD = (root) mean squared successive differences.

|  |  |  |  |
| --- | --- | --- | --- |
|  |  |  |  |
|  |  |  |  |
|  |  |  |  |
|  |  |  |  |
|  |  |  |  |
|  |  |  |  |
|  |  |  |  |
|  |  |  |  |
|  |  |  |  |
|  |  |  |  |
|  |  |  |  |
|  |  |  |  |
