# Supplementary material for: Variability and Influencing Factors of Suicidal Ideation in Adolescents—An Ecological Momentary Assessment Study
Source: Suicide Life Threat Behav. 2026 Nov 8;56(6):e70149. doi: 10.1111/sltb.70149 (PMC13745968; doi:10.1111/sltb.70149)
Supplement: Supplementary file 6 [file SLTB-56-0-s006.docx]

**Table S2**

*Repeated-Measures Correlations Among Suicidal Ideation Items and Subscales*

| Level | Variable 1 | Variable 2 | rᵣₘ | 95% CI | *p* |
| --- | --- | --- | --- | --- | --- |
| Item | Passive SI – Item 1 | Passive SI – Item 2 | .69 | .66–.71 | < .001 |
| Item | Active SI – Item 1 | Active SI – Item 2 | .68 | .65–.70 | < .001 |
| Item | Passive SI – Item 1 | Active SI – Item 1 | .61 | .58–.64 | < .001 |
| Item | Passive SI – Item 1 | Active SI – Item 2 | .49 | .46–.53 | < .001 |
| Item | Passive SI – Item 2 | Active SI – Item 1 | .59 | .56–.62 | < .001 |
| Item | Passive SI – Item 2 | Active SI – Item 2 | .55 | .52–.58 | < .001 |
| Subscale | Passive SI | Active SI | .67 | .65–.67 | < .001 |

*Note.* *r*_rm_ = repeated-measures correlation coefficient; SI = suicidal ideation. Passive SI – Item 1: 'Life is not worth living for me'; Passive SI – Item 2: 'There are more reasons to die than to live for me'; Active SI – Item 1: 'I think about taking my life'; Active SI – Item 2: 'I want to die'.
