# Supplementary material for: Variability and Influencing Factors of Suicidal Ideation in Adolescents—An Ecological Momentary Assessment Study
Source: Suicide Life Threat Behav. 2026 Nov 8;56(6):e70149. doi: 10.1111/sltb.70149 (PMC13745968; doi:10.1111/sltb.70149)
Supplement: Supplementary file 7 [file SLTB-56-0-s007.docx]

**Table S3**

*Intraclass Correlation for Suicidal Ideation Items*

| Item | ICC |
| --- | --- |
| Passive SI – Item 1 | 0.53 |
| Passive SI – Item 2 | 0.59 |
| Active SI – Item 1 | 0.48 |
| Active SI – Item 2 | 0.52 |

*Note.* ICC = intraclass correlation coefficient; SI = suicidal ideation. Passive SI – Item 1: 'Life is not worth living for me'; Passive SI – Item 2: 'There are more reasons to die than to live for me'; Active SI – Item 1: 'I think about taking my life'; Active SI – Item 2: 'I want to die'.
