# Supplementary material for: Variability and Influencing Factors of Suicidal Ideation in Adolescents—An Ecological Momentary Assessment Study
Source: Suicide Life Threat Behav. 2026 Nov 8;56(6):e70149. doi: 10.1111/sltb.70149 (PMC13745968; doi:10.1111/sltb.70149)
Supplement: Supplementary file 8 [file SLTB-56-0-s008.docx]

**Figure S2**

*Heat Maps*

Figure S2.2 Heat Map of Active Suicidal Ideation

Figure S2.1 Heat Map of Passive Suicidal Ideation

*Note.* The ratings of current passive and active are color-coded, with green indicating low levels, red indicating high levels, and grey indicating missing observations. Each row represents one participant, and each square corresponds to one rating. The x-axis displays the 49 prompts, vertical lines separate the days (day 1 to 7); the y-axis orders participants by their (R)MSSD in passive and active SI, respectively, with higher (R)MSSD values in the upper part of the heat map and lower (R)MSSD values in the lower part.
